# Evaluating wrapping alpine ski boots during on-snow carving

**DOI:** 10.3389/fspor.2023.1192737

**Published:** 2023-07-14

**Authors:** Eric C. Honert, Kathryn Harrison, Daniel Feeney

**Affiliations:** Performance Fit Laboratory, BOA Technology Inc., Denver, CO, United States

**Keywords:** biomechanics, wearable technology, plantar pressure, dorsal pressure, IMU

## Abstract

**Introduction:**

Alpine ski boots enable rapid and precise force transfer between skier and ski while carving. These boots are made of rigid plastic and fit tightly commonly through four buckles. Such a fit can improve speed and control but also pain and discomfort. In athletic footwear, alterations to the upper designed to wrap the foot improve performance during rapid changes of direction and during trail running. The purpose of this study was to systematically evaluate the performance and fit of two different ski boot shell closure mechanisms: a BOA closure and a Buckle closure.

**Materials and methods:**

This was a two-part study with 22 subjects performing on-mountain skiing and 10 of those subjects completing an in-laboratory pressure evaluation. Subjects skied in both boots three times each while data from inertial measurement units (IMUs) and plantar pressures were collected along with subjective data. In lab, static dorsal and plantar pressures were collected while the subjects flexed into the boots.

**Results:**

The BOA boots improved subjective and objective ski performance; qualitative carving scores were greater, likely through increasing the amount of normal force applied to the ski while turning. There were no differences in edge angles between the boots, as computed from IMUs. The BOA boot also reduced static peak plantar pressures in the rearfoot along with reducing overall static pressure on the dorsum as compared with the Buckle boot.

**Conclusions:**

This is the first study to systematically evaluate differences in ski boot closures. The improvements in carving performance in the BOA boot are supported by distinct differences in pressure distribution within each boot, which we speculate contributed to improved performance by reducing discomfort or pain while still facilitating effective force transfer.

## 1. Introduction

There are numerous alpine skiing forms such as carving, freestyle (mogul), and aerial maneuvers that are practiced at the competitive and non-competitive levels. Due to the repeatable nature of carving turns, they present an objective paradigm to assess skier performance outside of a race setting and to study the impact of various product features on skier performance. Alpine skiing performance during carving skiing in non-race settings can be parameterized with a set of force, angle, and time features that characterize the type of turn the skier is executing (1–4). Carving ski turns are characterized by skiers reaching high edge angles through large peak forces while arcing side-to-side down a ski slope, and advanced skiers increase both of these metrics compared to novice skiers (3, 5, 6). With the advent of accessible wearable technology, both measures of performance are easily obtainable via inertial measurement units (IMUs) (7, 8) and plantar pressure sensors (3, 8–11) with minimal impact on the skier.

Alpine ski boots are a critical piece of equipment enabling rapid and precise force transfer between a skier and a ski while carving. These boots are made of rigid plastic and fit tightly to match a skier's anthropometry in an attempt to minimize energy dissipation while skiing (12–14). For high-performance skiing, athletes tend to select the most rigid and snug-fitting boots possible. However, rigid, tight-fitting ski boots exert enough pressure to exceed blood pressure and cause numbness in the limb (15–18). Specifically, the compression of the plastic shell onto the user's foot caused by the boot Buckle creates local maxima in pressure, reducing circulation, which could impair performance and temperature regulation (18). Moreover, quantitative pressure distributions coupled with qualitative feedback suggest that better-fitting boots have lower peak pressures (19, 20). In low-cut footwear, lower peak dorsal pressures (21, 22) and lower dorsal contact areas result in more comfort (21). Similarly, lower peak plantar pressures are observed in footwear with comfortable orthotics (23, 24). On the other hand, higher-performing footwear increases plantar contact area (25). Such pressure measurements have been obtained in static scenarios that mimic an alpine boot try-on experience (19); however, there is an open question as to how systematic changes in alpine ski boot shell closures affect both plantar and dorsal pressures during such an experience.

The purpose of this study was to systematically evaluate the performance and fit of two different ski boot shell closure mechanisms, specifically a Buckle closure and a BOA closure. The BOA shell closure starts from a closure dial close to the ankle and routes a steel cable through guiding pulleys that are offset from one another (see Appendix Figure A1); in contrast, Buckle closure is directly parallel to the closure mechanism. The design intention of the BOA shell closure was to wrap around the foot. In athletic footwear, alterations in the fit of the product designed to wrap the foot result in improved performance during rapid changes of direction (26, 27) and trail running (25). We hypothesized that the BOA shell closure would improve skiing performance through a more uniform pressure distribution on the dorsum on the foot.

## 2. Materials and methods

**Figure 1:**
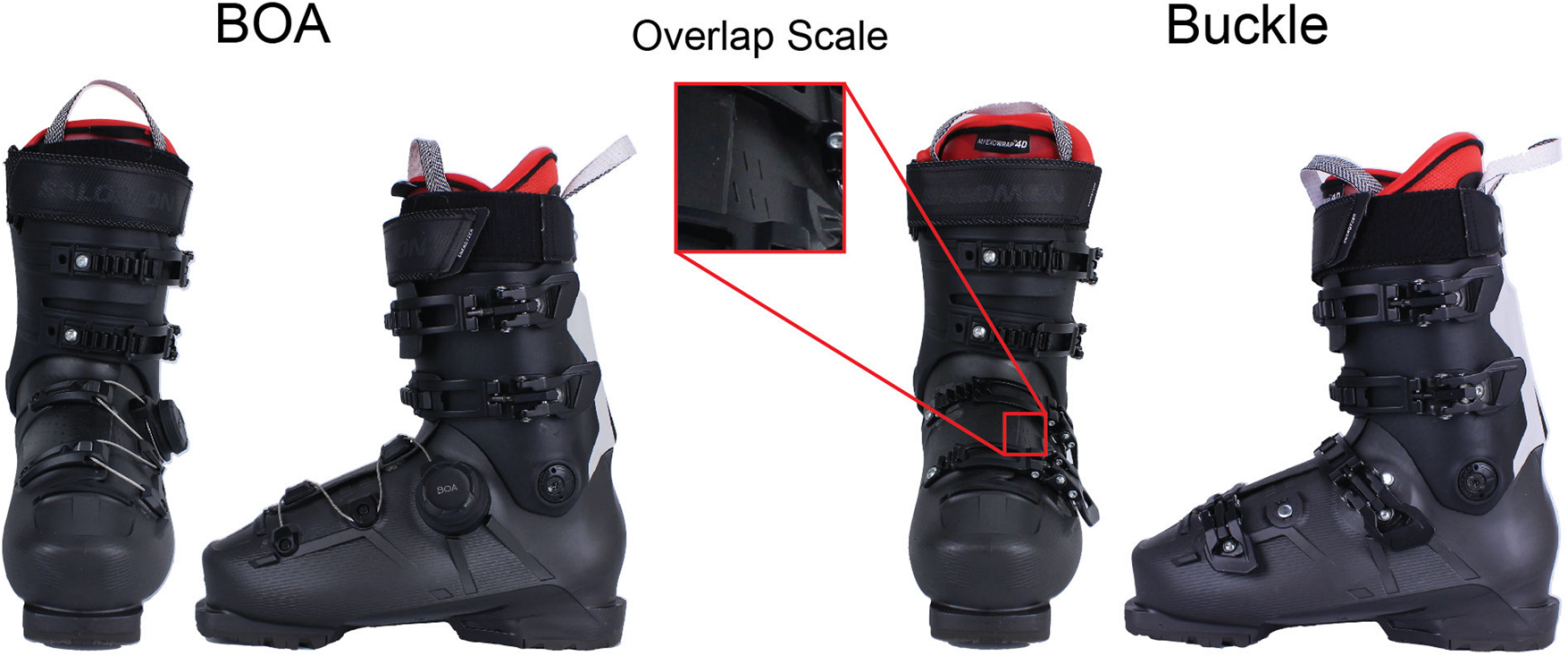
BOA (left) and Buckle (right) configurations of the Salomon S/Pro Supra Max. The Buckle configuration was originally produced with a BOA dial and was retrofitted with Buckles. Both boots have a scale to determine the overlap of the shell.

This study comprises two portions: on-mountain skiing and an in-laboratory pressure evaluation. In both locations, two ski boots (Salomon S/Pro Supra Max 120 Flex) that were identical aside from the shell (lower) boot closure system were tested: BOA and Buckle (Figure 1). All participants provided informed consent, and the testing was completed with IRB approval (IRB number: 22-BOAT-102).

### 2.1. Participants

We performed an *a priori* power analysis using the simr package in R, where we used simulations to estimate the number of subjects required to achieve 80% power using linear mixed-effects models for on-snow and in-laboratory variables (28). Based on preliminary data, we estimated the effect size in the laboratory to be twice that of the effect on-skiing due to the direct mechanical impact of closure on the foot. As a result, 22 male participants (height: 178 ± 6 cm, weight: 76 ± 9 kg, age: 40 ± 14 years) who were comfortable skiing experts and proficient in double black diamond ski runs reported that they knew and routinely practiced carving turns and wore size 26/26.5 or 27/27.5 ski boots to took part in the on-mountain protocol. Due to the larger estimated effect on dorsal pressure distribution, a subset of 11 skiers that participated in the on-mountain portion was subsequently invited to participate in the in-laboratory pressure evaluation. These 11 were chosen out of convenience (i.e., were able to transport themselves to the laboratory in a timely manner as written in our IRB). Participants were free of injury for the previous 6 months.

### 2.2. On-mountain protocol

All participants performed seven ski runs down a blue (intermediate) difficulty ski run with an average gradient of 23% at Eldora Ski Mountain, Nederland, CO, United States. The first run was used as a familiarization with the ski slope, and the subsequent six runs were performed as two sets of three runs in each boot type (BOA or Buckle—Figure 1) in a randomized order. Participants used their skis, with bindings adjusted as necessary to fit the study boots. Skiers were instructed to ski a similar line and make repeatable and aggressive carving turns alternating between left and right turns during each run. We opted not to utilize slalom poles or other marks in the snow as we did not want to influence the subject's preference for turn radius or comfortable skiing speed. The boots tested in this study are 120 flex, which is substantially more flexible than that of elite racing boots, so this protocol provides a more ecologically valid data collection setting. We measured normal force in three locations under both feet (heel, medial forefoot, and lateral forefoot) using plantar pressure insoles in the ski boots (100 Hz, Novel Loadsol-2, Munich, DEU, dimensions: 278 mm × 95 mm × 3.4 mm). A diagram of where the insoles were placed is in the Appendix. These insoles were shown to be repeatable and reliable during shod dynamic tasks as compared to force plates (29, 30). A trained experimenter placed the insoles on top of the ski boot foot bed (outside of the ski sock). The experimenter ensured that there was little movement in the insole when the subject slid their foot into the boot by placing double-sided tape on the insole and aiding the subject (should they need it) with donning the boot. Bilateral ski boot roll angles were computed from IMUs (±16 g and ± 2,000°/s at 1,125 Hz with 16-bit sensitivity and ±200 g at 1,600 Hz with 13-bit sensitivity, IMeasureU, Vicon, Denver, United States) fixed to the posterior longitudinal axis of the boots. The plantar force sensors transmitted data via Bluetooth to an iPhone placed in the subject's pocket. IMU data were stored on-board the device. Additionally, we measured the peak and average speed of participants with a GPS watch at 1 Hz (Suunto, Vantaa, Finland) and qualitative feedback for each boot. We extracted force, IMU, and GPS data from the top five to ten turns of each run to ensure clean and consistent data were compared across trials and conditions. Accordingly, data were segmented to be used only during the first 15 s of the run so we could ensure no obstacles or other skiers would interfere with the participant and similar turn locations would be compared across all trials.

### 2.3. In-laboratory protocol

Eleven participants came into the laboratory to measure static pressure on the dorsal and plantar aspects of their feet using capacitive insoles (XSENSOR, Calgary, CAN, sensor thickness: <2.0 mm). The dorsal pressure pad was a rectangular sensor with 180 sensing locations (18 rows with 10 columns, total dimension: 150 mm × 105 mm), and the plantar pressure had 235 sensing locations (total dimensions: 310 mm × 105 mm). A diagram of where these sensors were placed can be seen in the Appendix. The purpose of this data collection was to mimic the try-on experience consumers would ultimately undergo and explore relations between static pressure variables with on-mountain performance. Participants tried the boots on under three conditions: BOA-preferred tension, Buckle-preferred tension, and BOA with the same amount of shell overlap as Buckle. The preferred tension conditions were randomized between BOA and Buckle, and the BOA with the same amount of shell overlap as Buckle was the last. The overlap was recorded based on the shell overlap scale (Figure 1). This overlap was photographed for each condition to ensure that similar overlap was achieved between Buckle and BOA with the same amount of shell overlap as the Buckle. This last additional trial was present in the in-laboratory protocol and not on the mountain due to time constraints on the mountain to not overburden the subject's time nor induce fatigue. Subjects were instructed to flex into the boot repeatedly between conditions. Average dorsal and plantar pressure outcomes were computed from a 10-s trial.

### 2.4. Biomechanical outcomes

We used the IMU to compute peak edge angles. The procedure for the left IMU is as follows (the same procedure was followed with the right IMU except negating the roll angular velocity first). We first aggressively filtered the roll gyroscope with a second-order, zero-lag, low-pass Butterworth filtered at 0.5 Hz for turn segmentation (31, 32). Local maxima were utilized for the start/end of a turn. This time point corresponds to when the ski is parallel to the ground. All trials were manually inspected by a trained experimenter for anomalies. The original roll gyroscope signal was then filtered with a second-order, zero-lag, low-pass Butterworth filtered at 6 Hz to determine the edge angle (7). This filtered roll angular velocity was integrated between the start and end of the turn. A linear correction was used to account for signal noise. The local maxima (from the first half of the turn) was the maximum edge angle when the corresponding ski was the outside or downhill ski. The local minima (from the second half of the turn) was the maximum edge angle from when the corresponding ski was the inside or uphill ski.

All pressure trials were manually inspected by experimenters for anomalies before proceeding. Next, a bidirectional 0.5-Hz low-pass filter was applied to the summed insole force from all three regions (total force) separately for each foot to determine the indices of minima and maxima of force (similar to the IMU turn segmentation), approximately representing the apex of each turn. Next, we ensured the peak forces alternated between the left and right sides by recursively looking at each peak and removing any subsequent, false detections if the alternate side did not exhibit a peak between them. When advanced skiers are performing carving turns to the right, the peak force occurs under the downhill (or left) foot and vice versa for turning to the left. We then extracted force metrics of the downhill foot during each turn based on previous research on force production during ski carving turns (3, 33). Forces from each region were filtered with a bidirectional, 6-Hz, low-pass filter prior to any feature extraction. We extracted the peak total foot force and average total force of the downhill ski as these metrics were significantly different between intermediate and expert skiers (3). Peak forces were determined as the maximum value of the force in the respective region during a symmetrical 1-s window around each low-pass filter maxima index. In this same window, we computed the maximum uphill ski force. Previously, the mean uphill ski force during the turning phase has been shown to be higher in advanced skiers (3). We utilized the maximum uphill ski force as a substitute metric as we were not able to delineate the turning phase as has been previously described (3). Due to the importance of the rate of force production to performance in multiple sports (34–36) and the ability of alternative closure systems to alter this variable in agility movements (26), we also extracted the average rate of force production under the entire foot during each turn. The rate of force production was determined as the average instantaneous derivative of force from the previous minimum to the subsequent maximum for one foot. The time over this period was also computed to understand if changes in the rate of force development were due to differences in force or time. The average force was determined as the mean force from the previous minimum to the subsequent maximum for one foot.

For the in-lab portion, we calculated the peak pressure and contact area for the dorsal pressure, as these metrics have been associated with low-cut footwear comfort (21, 22). We also examined the mean pressure over the entire sensor, the standard deviation of pressure, and the total (summed) pressure for the dorsal region as these metrics have not been explored for dorsal pressures. For the plantar pressure, we examined peak pressures and contact areas in eight different regions: medial/lateral heel, medial/lateral midfoot, medial/lateral metatarsals, and medial/lateral toes. Peak pressures are associated with footwear fit and comfort (21, 23, 37).

All processing of biomechanical metrics was performed in Python (v3.9.7).

### 2.5. Subjective outcomes

Prior to the first ski run, the subjects reviewed the questionnaire. The questionnaire asked about carving performance, confidence, overall fit, forefoot fit, midfoot fit, heel fit, and cuff fit on an ordinal scale from 0 to 10. There was also a free-response section. After three laps in each boot, the subjects responded to the questions. For carving performance, confidence, and overall fit, 0 indicated the worst and 10 indicated the best. For specific foot regions (e.g., midfoot), 5 was perfect, 0 was too loose, and 10 was too tight. Additionally, subjects rated their exertion on each series of three runs on an ordinal scale from 0 (not tired) to 10 (exhausted).

### 2.6. Statistics

We utilized a linear mixed-effects model to evaluate on-mountain insole and IMU-based metrics. To accommodate the unequal number of observations for each dependent variable in each configuration, we used a linear mixed-effects model. This model encodes a random intercept for each subject and random slopes for each configuration (*Config*: BOA or Buckle), with fixed effects for turn direction (*TurnDirection*: left or right) and for trial number (*TrialNo*) for each biomechanical outcome (*Outcome*, Eq. 1). These additional fixed effects were added within the model as (1) the slope of the run was off camber such that greater forces were required when turning to the left and (2) snow conditions may not have been consistent between trials.

(1) Outcome∼Config+TurnDirection+TrialNo+(Config|Subject)

For the subjective outcomes and GPS speed, a separate linear mixed-effects model was utilized as there were fewer observations per subject (Eq. 2). This model only contained a random slope for each subject. The subjective data for the forefoot, midfoot, heel, and cuff were transformed such that 0 was optimal and 5 was too loose or too tight, as we were interested in understanding if the fit in each region of the boot was more “optimal” (i.e., closer to 5 on the original scale).

(2) Outcome∼Config+(1|Subject)

We evaluated the in-lab dorsal and plantar pressure outcomes using a third model (Eq. 3) as the amount of shell overlap between the preferred BOA condition and BOA at the Buckle overlap condition was not significantly different (as evaluated using Eq. 2). This third linear mixed-effects model contained a random intercept for each subject and the configuration was BOA or Buckle, similar to the other two models (Eqs. 1, 2). We combined both BOA conditions together and added the shell overlap as a fixed effect. Using such a model, we estimated the difference between the BOA and Buckle configurations *at a given overlap*, rather than making comparisons between three separate conditions.

(3) Outcome∼Config+Overlap+(1|Subject)

In the following, we present percent differences between the BOA and Buckle configurations. These differences were computed from the estimated marginal means from the respective models. All statistical analyses were performed in R Studio (version 4.1.2) with the LMER (38) and emmeans (39) packages. For all statistical tests, *α* was set to 0.05. These models (Eqs. 1–3) represent a natural way to model subject responses to footwear (40).

## 3. Results

One subject was excluded from all analyses (both on-mountain and in-laboratory) as fewer than five turns per trial were detected.

### 3.1. On-mountain

Subjects rated the carving, confidence, and overall fit higher in the BOA than in the Buckle (*p* < 0.01, Table 1). The subjective ratings of fit around the forefoot, midfoot, and heel were more optimal (i.e., rated closer to 5, *p *< 0.04). There was no difference in the rating of the cuff or the perceived exertion (*p* > 0.7).

**Table 1:** Subjective outcomes for the BOA and the Buckle boots.

	Exertion	Carving	Confidence	Overall Fit	Forefoot	Midfoot	Heel	Cuff
BOA	2.5 ± 1.8	8.5 ± 1.4	8.8 ± 1.2	8.0 ± 1.7	4.8 ± 1.7	5.1 ± 0.9	4.7 ± 1.0	5.0 ± 1.4
Buckle	2.5 ± 2.0	7.9 ± 1.6	8.2 ± 1.7	6.5 ± 2.1	5.8 ± 2.5	6.1 ± 2.2	4.6 ± 1.5	5.8 ± 1.2
*p*-value	1	0.004	0.01	0.003	0.02	0.004	0.04	0.7

Presented are study means ± standard deviations (*N* = 21). A linear mixed-effect model was used to understand statistical differences in the subjective outcomes. Note that data from the forefoot, midfoot, heel, and cuff were transformed prior to statistical analyses to understand if the fit in the region was closer to optimal (i.e., 5).

**Figure 2:**
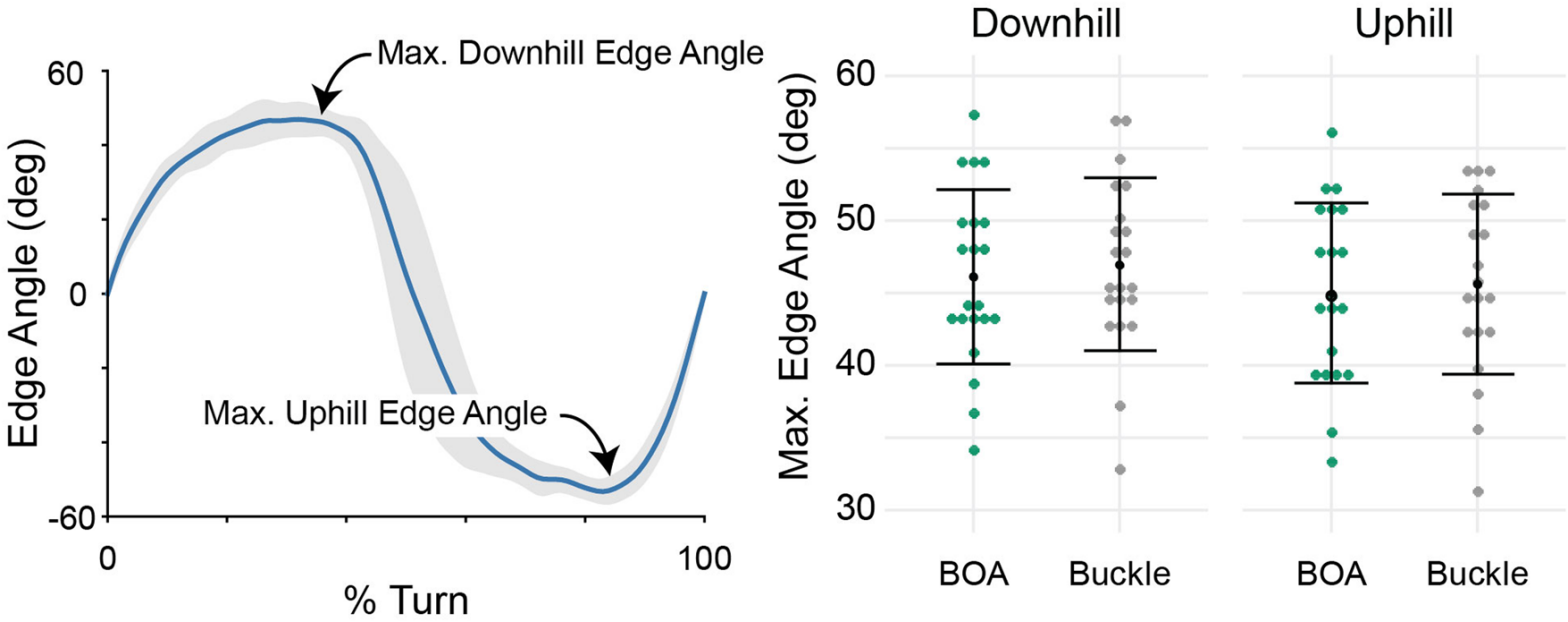
Estimated ski edge angles from a gyroscope. The time-continuous curve (left) is a representative mean (dark line) and standard deviation (shaded region) edge angle from one subject and one trial. The average maximum edge angles from each subject in each boot are shown in green (BOA) and gray (Buckle). Study means and standard deviations are represented in black (*N* = 21).

**Figure 3:**
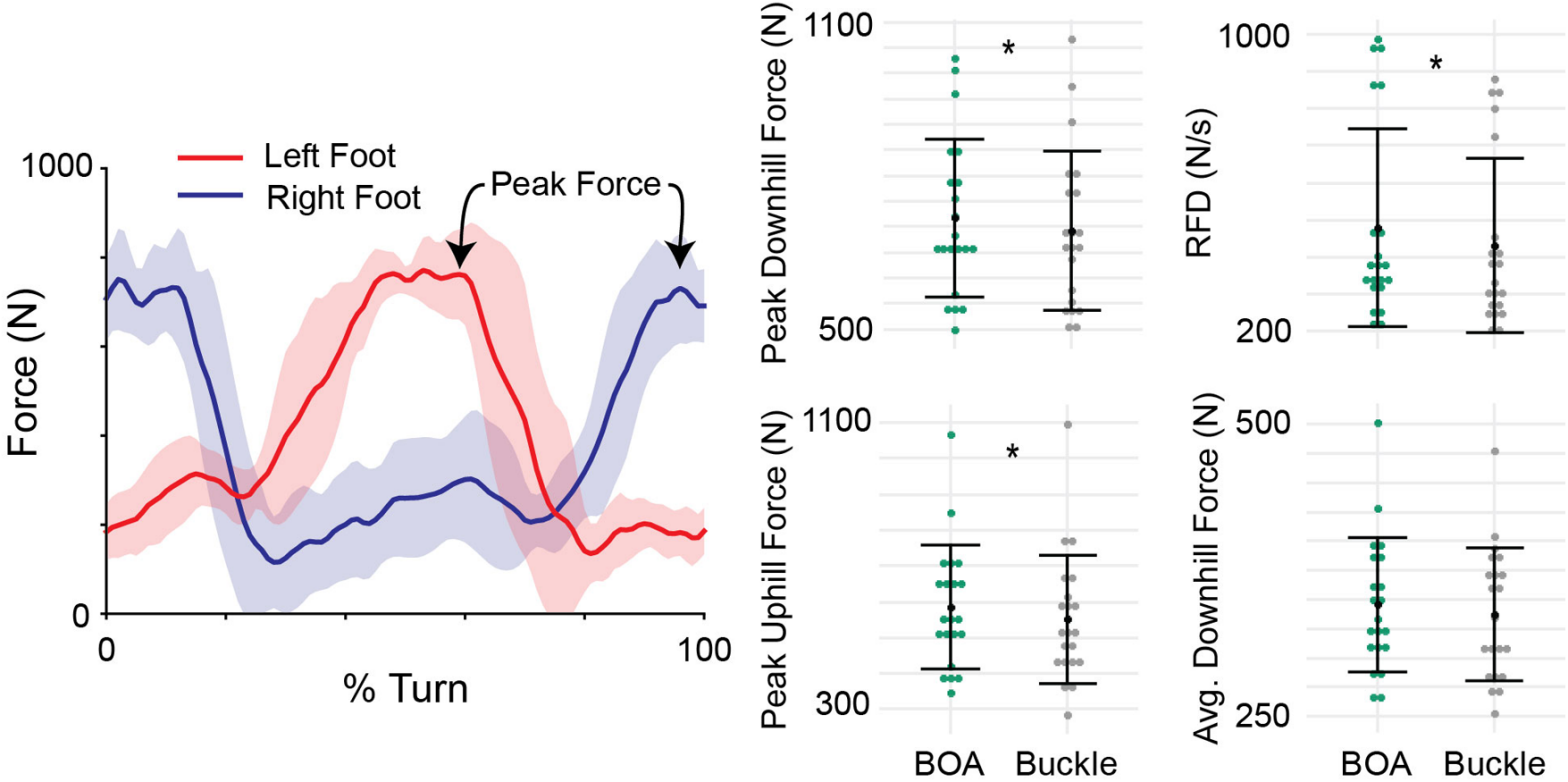
Normal force to the foot and affiliated outcome metrics. The time-continuous curve (left) is a representative mean (dark line) and standard deviation (shaded region) normal force from one subject and one trial. The average metrics from each subject in each boot are shown in green (BOA) and gray (Buckle) on the right. Study means and standard deviations are represented in black (*N* = 21). RFD is the rate of force development. Asterisks indicate significant differences between BOA and Buckle (*p *< 0.045).

Subjects’ top and average speeds did not differ between the conditions (*p *> 0.12). Additionally, the downhill and uphill edge angles did not differ between the boots (*p* > 0.11, Figure 2). Peak downhill ski force was on average 4% greater in the BOA configuration relative to Buckle (30 N, *p* = 0.045, Figure 3). The rate of force production under the downhill ski was on average 10% greater in BOA relative to Buckle (44 N/s, *p* = 0.002). Peak uphill ski force was on average 6% greater in BOA relative to Buckle (35 N, *p* = 0.038). Additionally, time to the peak ski force was on average 7% faster in BOA (0.13 s, *p* = 0.033). There were no differences in average force during turns (*p* = 0.33).

### 3.2. In-laboratory

**Figure 4:**
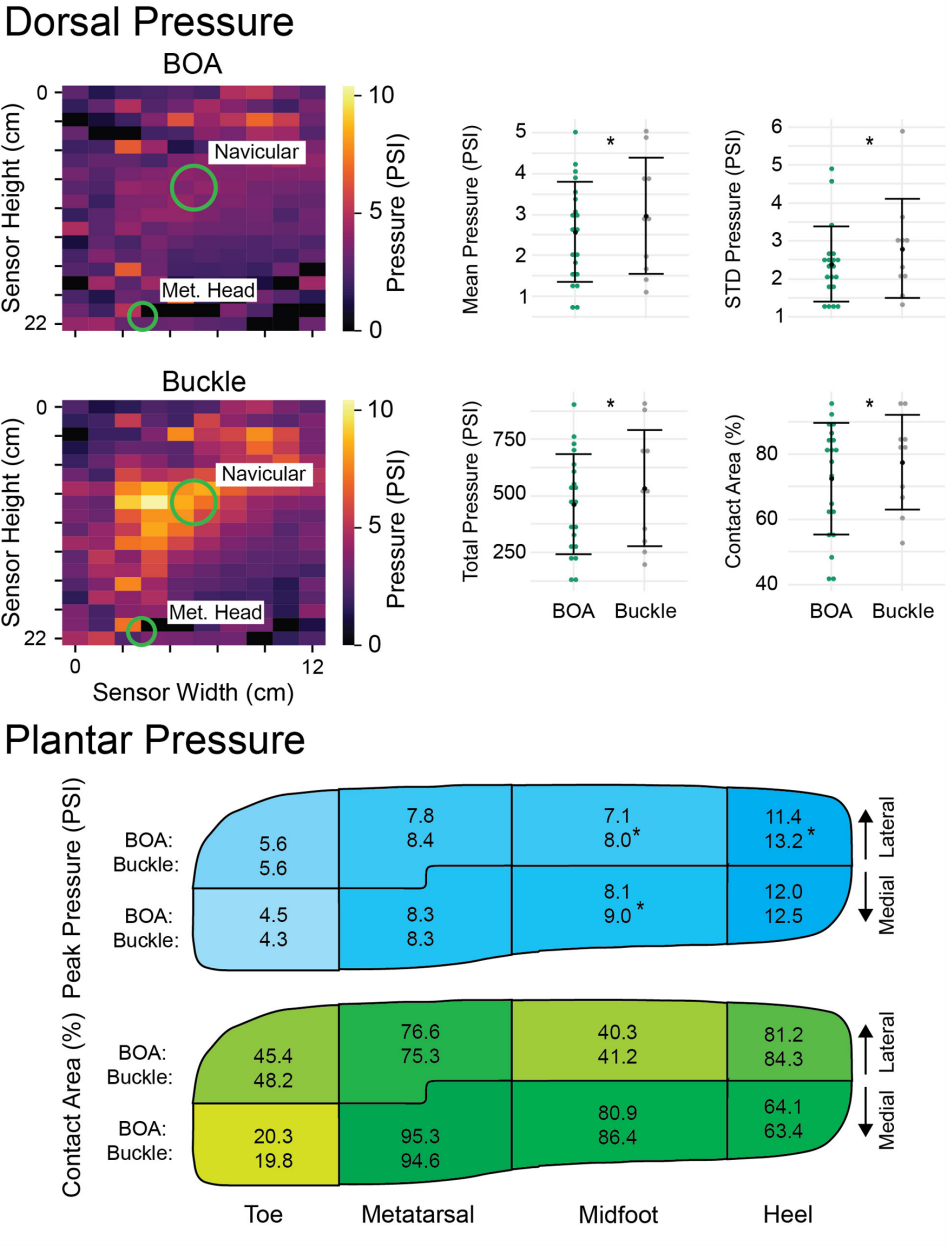
Study average dorsal and plantar static pressure outcomes. The preferred BOA tension and the BOA tension at the Buckle overlap were combined under BOA as there was no significant difference in the overlap between the preferred conditions. (Top) Example dorsal pressure for the BOA and Buckle ski boot with the same shell overlap (left) and associated outcome metrics (right). Each dot for the outcome metrics represents one subject. Asterisks indicate significant differences between the BOA and the Buckle (*p* < 0.013). (Bottom) Reported are estimated marginal means from the linear mixed-effects model with the BOA configuration appearing on the top above Buckle in the respective region. Asterisks indicate significant differences (*p* < 0.008).

In the BOA configuration, there was an average reduction of 12% in the mean dorsal pressure (*p* = 0.013, Figure 4) and an average reduction of 15% in the dorsal pressure standard deviation across the entire surface (*p* = 0.013). Additionally, there were average reductions of 13% (*p* = 0.013) and 6% (*p* = 0.0062) in the BOA for the total (summed) dorsal pressured and dorsal contact area, respectively. There was an average reduction of 16% in the peak dorsal pressure; however, this difference was not significant (*p* = 0.098). There was an average reduction of 14% (*p* = 0.001, Figure 4) in the BOA in the peak lateral heel pressure and 10% (*p* < 0.008) in the medial and lateral midfoot pressures in the BOA boot. There were no significant differences in medial nor lateral peak pressures in any other plantar region (*p* > 0.5) nor any significant differences in medial nor lateral contact areas in any plantar region (*p* > 0.09). All differences in static pressures between BOA and Buckle are provided at a given overlap. See the Appendix for results in a tabular form.

## 4. Discussion

This is the first study to systematically evaluate differences in ski boots, specifically ski boot shell closures, during on-snow skiing and static pressures. The BOA boots improved subjective and objective ski performance by improving qualitative carving scores and by increasing the amount of normal force applied to the ski while turning. The BOA boot also reduced peak plantar pressures in the rearfoot along with reducing overall pressure on the dorsum. Based on previous research combining subjective reports and objective data, this corresponds with a better overall fit along with a better fit in the forefoot, midfoot, and heel.

We explored a novel metric for ski research, the rate of force development, as this metric has been indicative of athletic performance (34–36) and is influenced by footwear closure (26). Previously, forces during ski carving have been evaluated between different levels of skiers (3), with greater peak and mean downhill forces associated with higher skiing levels. Yet, such forces are applied over time (not in isolation) as a skier progresses onto the edge of the ski. We observed that *both* peak force and time to peak force, and thus the rate of force development, improved with the BOA shell closure, indicating that athletes were obtaining the downhill ski edge faster and with better technique.

The improvements in carving performance in the BOA boot are supported by distinct differences in pressure distribution within each boot, which we speculate contributed to improved performance by improving comfort and facilitating effective force transfer. The dorsal region, specifically the instep, is a difficult region for ski boot fit (19). Clamping over the region with Buckles can result in loss of blood pressure to the foot, resulting in a decrease in temperature and the “cold leg” phenomenon (18). Here, we observed a significant reduction in several different dorsal pressure metrics. Previously, only peak dorsal pressures (21, 22, 41) and dorsal pressure contact areas (21) have been investigated with respect to footwear fit and comfort. While we did not observe a statistically significant reduction in peak dorsal pressure (there was an average reduction of 16% in the BOA configuration), we observed a reduction in the dorsal contact area that has been associated with improved comfort in low-cut footwear (21). The reduction in the standard deviation of the dorsal pressure provided by the BOA configuration means that there is a more even pressure distribution across the dorsum. This may be associated with the better wrapping that the BOA system provides over the Buckle that discretely tighten over two regions. This could be the reason why subjects rated the forefoot and midfoot fit more optimally. We also observed a reduction in the peak pressure in the lateral heel along with the midfoot. We hypothesize that this occurred due to the Buckle closure creating a non-uniform fit (as observed by higher dorsal pressure standard deviation), thus unevenly clamping the foot to the footbed. Regardless, such peak pressures have been linked to subjective comfort (23, 24), which may be why subjects rated the fit in the heel region more optimal.

In this study, we utilized three different statistical models to understand the differences between the BOA and Buckle boots. Due to the individual skill and foot morphology of each skier, we modeled all outcome variables with subject-specific intercepts (40) and used random slopes between conditions when justified by our experimental design and when we had enough samples in each condition to estimate the random parameters (42). For all models, we modeled outcome variables as a function of configuration. For on-mountain variables, it was necessary to include turn direction and trial number as these parameters substantially improved the goodness-of-fit of our models by explaining additional covariates such as the camber of the run and the changing quality of the snow, which often monotonically worsened over the course of a test. For in-lab pressure variables, we used boot overlap as a covariate to evaluate the impact of closure for a given level of boot overlap. In this way, we evaluated pressure distribution due to the mechanical differences in how each system closes the boot rather than from subjective differences in how participants would tension each boot. For all qualitative and in-lab pressure data, we did not have enough data in each condition to estimate random slopes for configuration, so we only fit the models with random intercepts for each participant.

There are several limitations to acknowledge with our study. First, we computed skier speed from wrist-based GPS, which computes speed based on the differentiation of the skier location at 1 Hz. Next, we estimated ski forces using pressure insoles due to their portability and minimal impact on the skier, so the skiers could use their own skis in the study. Previously, higher fidelity ground reaction forces, estimated via load cells, have differentiated between skier abilities (3). However, such systems displace the skier from their bindings and are several orders of magnitude heavier, which would have caused a compounding factor in systematically understanding how ski boot closures affect ski boot performance. Although thermal comfort is a concern with ski boots, we did not measure quantitative or qualitative measures of thermal comfort over the short period of time subjects were in ski boots. We utilized two different pressure measurement systems between on-snow (Novel LoadSol) and in-lab (XSENSOR). The LoadSol plantar pressure sensors had longer leads to the external data acquisition module such that the electronic connection point would not be strained while skiing. The XSENSOR system had a shorter lead to a similar module such that there was concern that skiing could put stress on the electronic connection point to the module, which may have resulted in equipment failure. We also did not measure the closure forces (43) from the Buckles or the BOA system. Finally, we only recorded in-lab pressure data from a subset of participants due to the difficulty of having participants report to both locations.

## 5. Conclusion

Alpine boots with a wrapping shell improved subjective and objective ski performance.

## Data Availability

The raw data supporting the conclusions of this article will be made available by the authors, without undue reservation.
