# Supplementary material for: Evaluating wrapping alpine ski boots during on-snow carving
Source: Front Sports Act Living. 2023 Jul 14;5:1192737. doi: 10.3389/fspor.2023.1192737 (PMC10379626; doi:10.3389/fspor.2023.1192737)
Supplement: Supplementary file 1 [file Datasheet1.pdf]

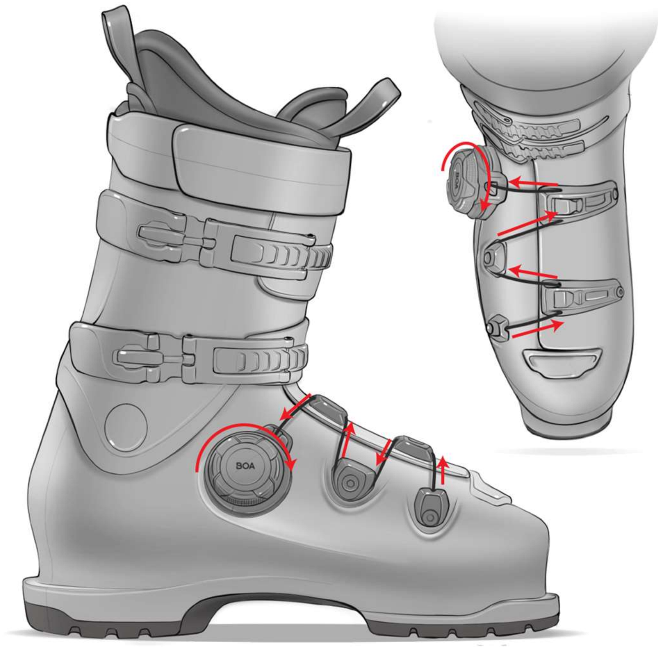

Figure A1. Side and top view of an alpine ski boot with a BOA shell closure. The arrow around the dial depicts the tightening direction. The arrows along the lace indicate the tightening path.

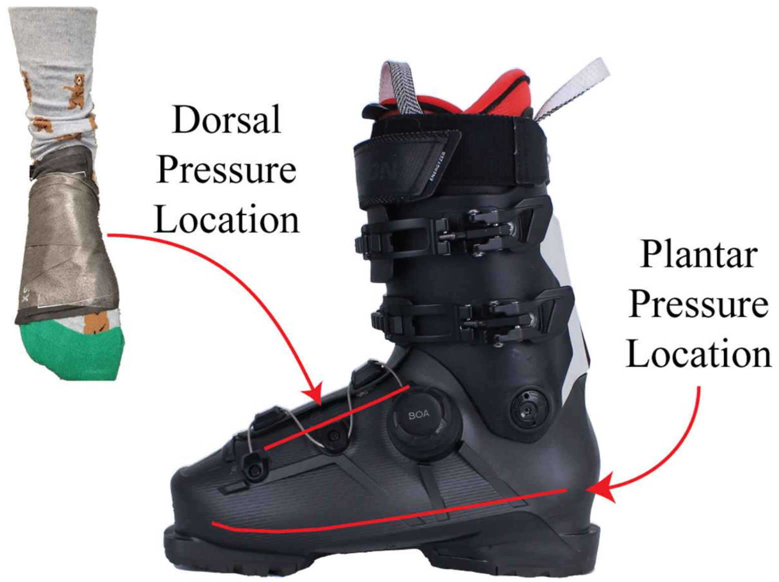

Figure A2. Example location for the plantar and dorsal pressure sensors within the boot. The dorsal pressure was only recorded which subjects were statically flexing the boot.

Table A1. Dorsal pressure static outcomes at a given shell overlap. Presented are estimated marginal means and standard deviations from a linear mixed effect model. Asterisks indicate significant differences between the BOA and Buckle shell at a given overlap (*p*<0.013).

	Mean Pressure (PSI)	Max Pressure (PSI)	Total Pressure (PSI)	SD Pressure (PSI)	Contact Area (%)
BOA	2.6±1.4*	12.7±5.6	464±248*	2.39±1.1*	72.7±16.8*
Buckle	3.0±1.4*	15.2±6.2	532±253*	2.8±1.1*	77.2±17.1*

Table A2. Plantar pressure static outcomes at a given shell overlap. Presented are estimated marginal means and standard deviations from a linear mixed effect model. Asterisks indicate significant differences between the BOA and Buckle shell at a given overlap (*p*<0.008).

	Medial Heel	Lateral Heel	Medial Midfoot	Lateral Midfoot	Medial Metatarsal	Lateral Metatarsal	Medial Toe	Lateral Toe
	Peak Plantar Pressure (PSI)							
BOA	12.0±2.7	11.4±4.3*	8.1±3.6*	7.1±2.4*	8.3±3.2	7.8±5.4	4.5±2.4	5.6±2.4
Buckle	12.5±2.9	13.2±4.4*	9.0±3.6*	8.0±2.4*	8.3±3.4	8.4±5.8	4.3±2.5	5.6±2.6
	Contact Area (%)							
BOA	64.1±2.7	81.2±8.4	80.9±19.8	40.3±20.6	95.3±9.7	76.6±12.8	20.3±9.4	45.4±20.4
Buckle	63.4±3	84.3±9.0	86.4±20.9	41.2±21.5	94.6±11.3	75.3±13.4	19.8±10.3	48.2±22.1
